# *Chromobacterium violaceum* Pathogenicity: Updates and Insights from Genome Sequencing of Novel *Chromobacterium* Species

**DOI:** 10.3389/fmicb.2017.02213

**Published:** 2017-11-10

**Authors:** Juliana H. Batista, José F. da Silva Neto

**Affiliations:** Departamento de Biologia Celular e Molecular e Bioagentes Patogênicos, Faculdade de Medicina de Ribeirão Preto, Universidade de São Paulo, Ribeirão Preto, Brazil

**Keywords:** *Chromobacterium violaceum*, *Chromobacterium* species, genome sequencing, comparative genomics, pathogenicity island, type III secretion system

## Abstract

*Chromobacterium violaceum* is an abundant component of the soil and water microbiota in tropical and subtropical regions around the world. For many years, it was mainly known as a producer of violacein and as a reporter for the discovery of quorum sensing molecules. However, *C. violaceum* has recently emerged as an important model of an environmental opportunistic pathogen. Its high virulence in human infections and a mouse infection model involves the possession of several predicted virulence traits, including two type III secretion systems (T3SSs). In this article, in addition to providing an update on the new clinical cases of human *C. violaceum* infections, we will focus on recent advances in understanding the molecular mechanisms regarding *C. violaceum* pathogenesis. It has been demonstrated that the *C. violaceum* Cpi-1 T3SS plays a pivotal role in interaction with host cells. It is required for the secretion of effector proteins and is the agonist recognized by the Nod-like receptor CARD domain-containing protein 4 (NLRC4) inflammasome from innate immune cells. Pyroptosis and its release of hepatocytes for killing by neutrophils are key events required for the clearance of *C. violaceum*. Given the prominent role of T3SSs in *C. violaceum* virulence, we examine their occurrence in the *Chromobacterium* genus, taking advantage of several draft genome sequences of *Chromobacterium* species that have recently become available. Our finding that the Cpi-1 T3SS is widespread among *Chromobacterium* species points toward the pathogenic potential of this genus for humans or to novel roles of the T3SS in the interaction of *Chromobacterium* species with other organisms.

## Introduction

For many years, studies on *Chromobacterium violaceum* have been focused on investigating small molecules of biotechnological interest derived from its secondary metabolism, while aspects related to the pathogenicity of *C. violaceum* have been neglected. In fact, there are many reviews describing the biotechnological and pharmacological importance of *C. violaceum* and its secondary metabolites, mainly the purple pigment violacein (Durán and Menck, 2001; Durán et al., 2007, 2016), but none have focused on the mechanisms of *C. violaceum* virulence. However, this situation has changed in recent years, with numerous works advancing toward revealing multiple facets of the biology of *C. violaceum* and its interaction with mammalian hosts (Miki et al., 2010, 2011; Maltez et al., 2015; Previato-Mello et al., 2017). In this work, we summarize recent advances in the knowledge of the pathogenesis of *C. violaceum* infections and update the scenario regarding clinical cases and deaths caused by *C. violaceum*. Moreover, we evaluate the presence and genomic organization of the genes encoding type III secretion systems (T3SSs) in members of the *Chromobacterium* genus.

### Overview of the *Chromobacterium* Genus

*Chromobacterium* is a genus of soil- and freshwater-associated Gram-negative bacteria within the *Neisseriaceae* family of Betaproteobacteria. Despite its saprophytic, free-living lifestyle, the species type of the genus, *C. violaceum*, has been associated with infections in humans and other animals (Durán and Menck, 2001; Yang and Li, 2011). One particular characteristic of this genus is the production of violacein, a purple pigment for which the synthesis is regulated by quorum sensing (Mcclean et al., 1997). However, non-pigmented isolates have also been identified (Sivendra and Tan, 1977). Violacein is a pigment with high biotechnological interest due to its *in vitro* activity against bacteria, fungi, protozoa, viruses, and tumor cells (Durán et al., 2007, 2016). Environmental isolates of *Chromobacterium* have the potential to be used in other biotechnological applications, including biocontrol of plant diseases caused by insect pests (*Chromobacterium* sp. strain C-61) (Kim et al., 2014), prevention of disease transmission by *Anopheles gambiae* and *Aedes aegypti* mosquitoes (*Chromobacterium* sp. Csp_P) (Ramirez et al., 2014), hydrogen cyanide-mediated gold recovery from electronic waste (Tay et al., 2013), and production of the anti-tumoral depsipeptide FR901228 (*C. violaceum*) (Durán and Menck, 2001; VanderMolen et al., 2011).

Although *C. violaceum* has been recognized as the single species of the *Chromobacterium* genus for a long time, nine novel species have been proposed since 2007: *C. subtsugae* (Martin et al., 2007), *C. aquaticum* (Young et al., 2008), *C. haemolyticum* (Han et al., 2008), *C. piscinae* (Kämpfer et al., 2009), *C. pseudoviolaceum* (Kämpfer et al., 2009), *C. vaccinii* (Soby et al., 2013), *C. amazonense* (Menezes et al., 2015), *C. alkanivorans* (Bajaj et al., 2016), and *C. rhizoryzae* (Zhou et al., 2016). Additionally, the great genetic variability found in *Chromobacterium* isolates collected from distinct tropical regions (Hungria et al., 2005; Lima-Bittencourt et al., 2007) supports the trend to attempt to recognize novel species. Most of the *Chromobacterium* species were isolated from environmental samples (mainly from water, soil, and rhizosphere) and have not yet been associated with human infections (**Table 1**). Exceptions include *C. violaceum*, isolated from both environmental and clinical samples and associated with several cases of fatal infections (Yang and Li, 2011), and *C. haemolyticum*, isolated from a patient’s sputum culture (Han et al., 2008) and associated with a human case of bacteremia (Okada et al., 2013). With respect to violacein production, the non-purple species of this genus are *C. aquaticum*, *C. haemolyticum*, *C. alkanivorans*, and *C. rhizoryzae* (**Table 1**).

**Table 1:** Summary of the *Chromobacterium* species and selected strains with genome sequences available.

Species/strain with published genome^a^	Isolation source	Biological interaction	Colony color	Reference of genome sequencing
*C. violaceum*/strains ATCC 12472 and CV017 (derived from ATCC 31532)	Soil and water worldwide; human infections	Human and animal pathogen	Violet	Vasconcelos et al., 2003; Wang et al., 2016
*C. subtsugae*/strains PRAA4-1; MWU12-2387; MWU3525; MWU2576; and MWU2920	Forest soil and rhizosphere in United States	Toxic to insect larvae	Violet	Vöing et al., 2015b, 2017; Blackburn et al., 2016
*C. aquaticum*/strain CC-SEYA-1	Spring water in Taiwan	Non-described	Tan	Soby, 2017a
*C. haemolyticum*/strain T124	Sputum from a patient in United States; bacteremia in Japan	Human pathogen	Gray	Miki and Okada, 2014
*C. piscinae*/strain ND17	Pond water in Malaysia	Non-described	Violet	Chan and Yunos, 2016
*C. pseudoviolaceum*/strain LMG 3953T	Unclear	Non-described	Violet	Soby, 2017b
*C. vaccinii*/strains MWU205 and MWU328	Soil and rhizosphere in United States	Toxic to insect larvae	Violet	Vöing et al., 2015a
*C. amazonense*	River water in Brazil	Non-described	Violet	–
*C. alkanivorans*	Contaminated soil in India	Degradation of halogenated alkanes	Tan	–
*C. rhizoryzae*	Rhizosphere in China	Inhibition of fungal pathogens	Tan	–
*Chromobacterium* sp./strain C-61	Rhizosphere in Korea	Inhibition of fungal pathogens		Kim et al., 2011


*^a^All 10 proposed *Chromobacterium* species are shown. In respect to genome sequence availability, we listed only the *Chromobacterium* strains whose genome sequence were published. A more comprehensive list of *Chromobacterium* genomes publicly available can be found in the NCBI GenBank database (https://www.ncbi.nlm.nih.gov/genome/?term=chromobacterium).*

### Updates Regarding Clinical Reports of *Chromobacterium violaceum* Infections

Although rare, human infections with *C. violaceum* are associated with high mortality rates; bacteria spreading rapidly to several organs, especially the liver, lungs, and spleen; and life-threatening sepsis (Sneath et al., 1953; Durán and Menck, 2001). The main clinical manifestations are fever, abdominal pain, skin lesions, and formation of metastatic abscesses. The most common route of transmission involves the exposure of wounds and traumatic lesions to soil and water containing *C. violaceum* (Martinez et al., 2000; Durán and Menck, 2001; Baker et al., 2008; Ansari et al., 2015). Due to the rapid clinical course of the chromobacteriosis, one important predisposing risk factor in *C. violaceum* infections is inappropriate antimicrobial therapy. It has been reported that *C. violaceum* is resistant to several antibiotics, mainly to some beta-lactams, but it is sensitive to others, such as carbapenems and quinolones (Aldridge et al., 1988). Indeed, most of the treatments which were successful in controlling infections involved the use of the antibiotics ciprofloxacin and meropenem (Nanayakkara et al., 2008; Ke et al., 2012; Campbell et al., 2013; Pant et al., 2017).

The last comprehensive compilation of human cases of *C. violaceum* infection analyzed 106 patients with *C. violaceum* infections between 1952 and 2009 (Yang and Li, 2011). Here, our update on the published clinical reports of *C. violaceum* infections (searching the PubMed database from 2010 to 2017) reveals that infections due to *C. violaceum* are still rare and are associated with high mortality (Supplementary Table S1). Overall, there are less than 150 published clinical reports describing human *C. violaceum* infections. During the period of our analysis, 23 new cases of human infection were reported, with a mortality rate of 35% (eight fatal cases) (Supplementary Table S1). This is a smaller value than what was seen at the time of the last update (53%) (Yang and Li, 2011). The reduction in the rate of fatal cases could be attributed to an improvement in antibiotic administration or better diagnostics. The tendency for the distribution of cases to be worldwide was maintained in our compilation, despite the most fatal cases having been described in developing countries (Supplementary Table S1). Moreover, recent reports of *C. violaceum* infection found that, in addition to systemic infections, this bacterium causes urinary tract infections and pneumonia in hospital environments, which raises concerns about its potential as a nosocomial pathogen (Hagiya et al., 2014; Swain et al., 2014; Pant et al., 2015, 2017).

In addition to *C. violaceum*, another species of potential medical interest in the same genus is *C. haemolyticum* (**Table 1**). Although only a few cases of human *C. haemolyticum* infection have been reported so far, and none of them were fatal, this bacterium has shown remarkable hemolytic activity against human and sheep erythrocytes (Han et al., 2008; Okada et al., 2013). It has several uncharacterized potential virulence factors, as predicted from the draft genome sequence of one clinical isolate (Miki and Okada, 2014). Interestingly, *C. haemolyticum* isolates collected from a tropical freshwater lake exhibited strong beta-hemolytic activity and high resistance to beta-lactam antibiotics, as observed in clinical isolates (Lima-Bittencourt et al., 2011).

### Molecular Pathogenesis of *C. violaceum* Infections

The sequencing of the complete genome of *C. violaceum* strain ATCC 12472 shed light on the virulence mechanisms of this bacterium by revealing the presence of many predicted virulence factors (Vasconcelos et al., 2003). The most remarkable of these predicted virulence factors was the type III secretion system (T3SS), which is a needle-like multiprotein complex that injects various bacterial effectors into host cells (Galán et al., 2014). Surprisingly, genomic data have revealed that *C. violaceum* has two T3SSs whose genes were clustered in *Chromobacterium* pathogenicity islands 1 and 2 (Cpi-1 and Cpi-2) (Vasconcelos et al., 2003; Alves de Brito et al., 2004). These islands were located next to each other on the chromosome, but while the genes from Cpi-2 were all grouped together, some genes from Cpi-1, encoding the needle complex, were located distantly from Cpi-1, in a cluster called the Cpi-1a (Betts et al., 2004). *C. violaceum* Cpi-1/1a and Cpi-2 resemble the well-characterized *Salmonella* pathogenicity islands Spi-1 and Spi-2 (Betts et al., 2004).

In 2010, it was demonstrated that deletion of genes from Cpi-1/1a, but not from Cpi-2, causes a profound reduction in *C. violaceum* virulence in a mouse model of infection, positioning Cpi-1/1a as the major determinant for *C. violaceum* pathogenicity (Miki et al., 2010). In addition, the capacity of *C. violaceum* to cause fulminant hepatitis in mice through the induction of cytotoxicity and cell death in hepatocytes was shown to be dependent on the Cpi-1/1a-encoded T3SS (Miki et al., 2010). Despite of the absence of a clear requirement for Cpi-2 for systemic infection by *C. violaceum* (Miki et al., 2010), more studies are necessary to understand the role of this T3SS in the interaction of *C. violaceum* with host cells. Cpi-2 could be involved in the survival of *C. violaceum* within macrophages, as described for the *Salmonella* Spi-2 system (Hensel, 2000).

Further investigations demonstrated that the repertoire of Cpi-1/1a-encoded T3SS effectors translocated into hepatocytes includes at least 16 effector proteins, but the role of the most of them have yet to be determined (Miki et al., 2010, 2011). The authors discovered that one of these effectors, called CopE, plays a key role in *C. violaceum* invasion of non-phagocytic epithelial cells and is required for *C. violaceum* virulence in mice (Miki et al., 2011). This study demonstrated that CopE acts as a guanine exchange factor (GEF) that activates Rac1 and Cdc42 in HeLa cells, resulting in the induction of actin rearrangement. Consequently, this promotes *C. violaceum* invasion of non-phagocytic cells (Miki et al., 2011). Interestingly, it has been recently discovered that *C. violaceum* escapes from the phagosome to the cytosol in epithelial cells by a mechanism involving CipC, a translocon apparatus protein of the Cpi-1 T3SS (Du et al., 2016).

The roles of transcriptional regulators in *C. violaceum* virulence have been investigated. For instance, a study based on expression and mutagenesis analyses of five putative regulators located within the Cpi-1 and Cpi-2 islands (CilA, CivF, ArmR, CsrB, and CsrC) revealed that CilA is the master transcriptional activator of most of the Cpi-1/1a genes (Miki et al., 2011). This is consistent with the previous finding that a *cilA*-mutant strain was fully attenuated for virulence in mice (Miki et al., 2010). The signals that turn on the expression of the CilA-regulated genes in *C. violaceum*, including the Cpi-1/1a genes, remain largely unknown. More recently, it has been reported that the MarR family transcriptional regulator OhrR is important for the virulence of *C. violaceum* in mice (Previato-Mello et al., 2017). In *C. violaceum*, OhrR is a sensor of organic hydroperoxides that regulates the expression of a few genes related to antioxidant defense, synthesis of cyclic di-GMP, and the production of virulence-related, secreted enzymes (da Silva Neto et al., 2012; Previato-Mello et al., 2017). Finally, the involvement of a quorum sensing system in the ability of *C. violaceum* to kill *Caenorhabditis elegans* has been determined (Swem et al., 2009), allowing this nematode to be used as an alternative model for identifying virulence genes of *C. violaceum*.

An elegant study demonstrated that the molecular detection of *C. violaceum* by human macrophages involves the recognition of the Cpi-1a T3SS needle protein CprI by the NAIP protein; human NAIP recognizes CprI and promotes Nod-like receptor CARD domain-containing protein 4 (NLRC4) inflammasome oligomerization, which is followed by caspase-1 activation and pyroptosis (Zhao et al., 2011). Subsequent investigations using a murine model revealed that *C. violaceum* infection is promptly controlled in healthy mice by the NLRC4 inflammasome via two pathways that release bacteria from intracellular niches: pyroptosis and Natural Killer (NK) cell cytotoxicity (Maltez et al., 2015). These mechanisms eject the intracellular bacteria from macrophages and hepatocytes and expose them to the action of neutrophils (Maltez et al., 2015). In fact, neutrophil deficiencies in NADPH oxidase, as seen in patients with chronic granulomatous disease (CGD), drastically increase susceptibility to *C. violaceum* infections (Segal et al., 2003; Yang and Li, 2011; Maltez et al., 2015). In addition, it was verified that knockout mice in these immune system pathways are extremely susceptible to infection by *C. violaceum* and *Burkholderia thailandensis*, two CGD-associated pathogens (Maltez et al., 2015). Therefore, a hypothesis was proposed, stating that inflammasomes evolved as a form of defense against infection due to environmental bacteria with virulence traits that did not evolve with vertebrate hosts (Maltez and Miao, 2016).

### Prevalence of T3SSs in the *Chromobacterium* Genus

Bacterial T3SSs have been demonstrated to be key determinants of virulence for many Gram-negative plant and animal pathogens via delivery of effector proteins into the cytosol of host eukaryotic cells (Deng et al., 2017). Gene clusters encoding T3SSs are also found in genomes of non-pathogenic bacteria, and the roles of these T3SSs are not restricted to pathogenesis, but seem to include other processes during interactions involving bacteria and their hosts in diverse ecological contexts (Nazir et al., 2017). As mentioned above, earlier genome sequencing of the strain type *C. violaceum* ATCC 12472 revealed the presence of two T3SSs (Cpi-1/1a and Cpi-2), of which, Cpi-1/1a is absolutely required for virulence (Vasconcelos et al., 2003; Miki et al., 2010). Recently, several draft genome sequences of *Chromobacterium* species were published (**Table 1**) and many other have become publicly available (39 *Chromobacterium* genomes, as searched on 15 June, 2017 in the NCBI GenBank database), allowing for a detailed inspection of the occurrence and organization of T3SSs in members of the *Chromobacterium* genus and among strains of the same species.

**FIGURE 1:**
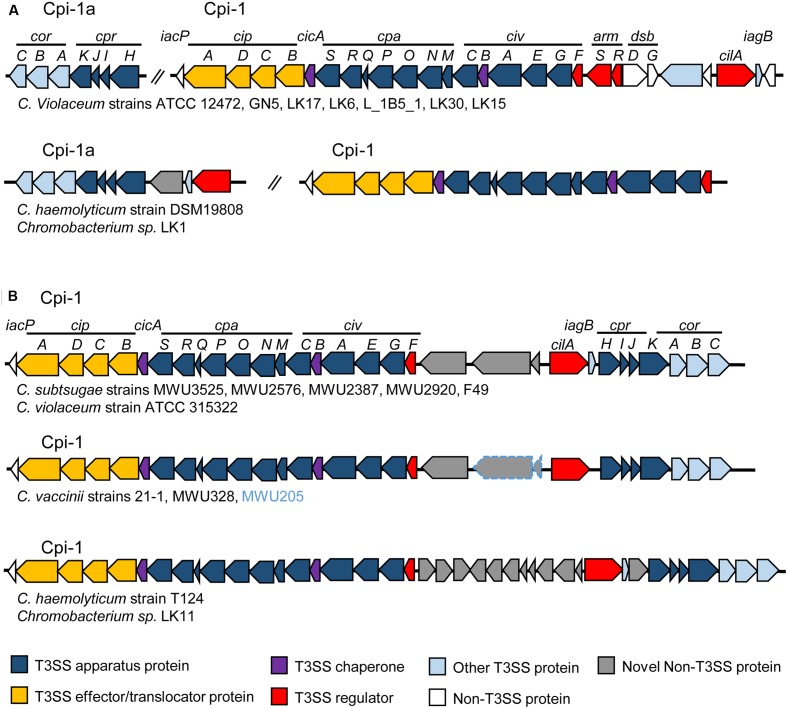
Genomic organization of *Chromobacterium* pathogenicity island 1 (Cpi-1) in members of the *Chromobacterium* genus. The T3SS genes are either split into two gene clusters (Cpi-1/1a) **(A)**; or grouped together (Cpi-1) on the chromosome **(B)**. Comparison was performed using T3SS gene clusters from *C. violaceum* ATCC 12472 (Cpi-1a, CV2417-CV2423 and Cpi-1, CV2615-CV2642) as previously annotated (Betts et al., 2004). Genes are colored according to functional category (genes in gray are that absent in *C. violaceum* ATCC 12472). For *C. vaccinii*, the two genes surrounded in blue are present only in MWU205 strain.

We performed such an analysis by searching for genes of the Cpi-1/1a- and Cpi-2-encoded T3SSs of *C. violaceum* ATCC 12472 in the draft genome sequences of 22 *Chromobacterium* species/strains (Supplementary Table S2), using tools available in the Integrated Microbial Genomes and Microbiome (IMG/M) system (Chen et al., 2016). Some interesting findings arose from this analysis (Supplementary Table S2 and **Figure 1**): (i) the widespread occurrence of the Cpi-1/1a T3SS in the *Chromobacterium* genus, since its absence was observed only in *C. piscinae* (Supplementary Table S2); (ii) the existence of two genomic organizations for the Cpi-1/1a genes, which were found either as two separated gene clusters (Cpi-1 and Cpi-1a, as seen in **Figure 1A**, for instance in *C. violaceum* strains), or as a single cluster of contiguous genes (Cpi-1, as seen in **Figure 1B**, for instance in *C. subtsugae* strains); and (iii) the narrow distribution of Cpi-2 in the *Chromobacterium* genus, since the occurrence of most Cpi-2 genes was restricted to *C. piscinae* and *C. vaccinii* (Supplementary Table S2). These data support the hypothesis that the presence of Cpi-1 is ancient in the *C. violaceum* genome (and perhaps in the *Chromobacterium* genus) and that Cpi-2 was acquired more recently (Betts et al., 2004).

## Concluding Remarks and Perspectives

In recent years, we have begun to learn how *C. violaceum* causes severe infection in mammalian hosts, despite its evolution as an environmental free-living bacterium. Evolutionarily, the most relevant event that makes it an opportunistic pathogen is very likely to be the acquisition and/or maintenance of Cpi-1, a pathogenicity island containing the T3SS, which is essential for *C. violaceum* virulence. This T3SS is important for the pathogenesis of *C. violaceum* because it causes damage to hepatocytes and promotes the invasion of non-phagocytic cells. Also, it is the signal that triggers activation of the NLRC4 inflammasome, resulting in an effective clearance of the infection by the innate immune system. The widespread occurrence of intact Cpi-1 in many *Chromobacterium* species (most of them isolated from environmental sources and yet not associated with human infection) raises questions about the potential of these species to be pathogenic for humans. An alternative hypothesis is that the Cpi-1 T3SS contributes to the interaction of *Chromobacterium* with other organisms. In fact, several *Chromobacterium* species have been isolated from the roots of plants and are able to suppress plant disease by killing insect larvae or antagonize fungal pathogens. Future studies involving the T3SSs and other virulence factors of *C. violaceum* will contribute to a better understanding of the pathogenesis of this rare but deadly human pathogen.

## Author Contributions

JB and JdS conceived the idea, wrote the manuscript, and prepared the figures and tables. JB performed the update regarding clinical reports of infections. JdS performed the analysis of prevalence and organization of T3SS genes.

## Conflict of Interest Statement

The authors declare that the research was conducted in the absence of any commercial or financial relationships that could be construed as a potential conflict of interest.
