# Supplementary material for: Chromobacterium violaceum Pathogenicity: Updates and Insights from Genome Sequencing of Novel Chromobacterium Species
Source: Front Microbiol. 2017 Nov 10;8:2213. doi: 10.3389/fmicb.2017.02213 (PMC5686120; doi:10.3389/fmicb.2017.02213)
Supplement: Supplementary file 1 [file Table_1.docx]

| **Countries** | **Nº of people infected** | **Mode of infection** | **Antibiotic therapy** | **Number of deaths** | **Reference(s)** |
| --- | --- | --- | --- | --- | --- |
| Vietnam | 1 | Skin wound | Meropenem | 0 | Campbell et al., 2013; |
| Nepal | 6 | Urinary tract infection, trauma, skin wound | Ofloxacin, meropenem, ciprofloxacin | 1 | Pant et al., 2017; Pant et al., 2015; Ansari et al., 2015; Parajuli et al., 2016 |
| Cambodia | 1 | Skin wound | Imipenem | 0 | Ke et al., 2012 |
| Solomon Islands | 1 | Skin wound | Meropenem | 0 | Meher-Homji et al., 2017 |
| Congo | 1 | - | Ceftriaxone and gentamicin | 1 | Bottieau et al., 2015 |
| India | 8 | Urinary tract infection, trauma, skin wound | Ciprofloxacin | 4 | Karthik et al., 2012; Kaniyarakkal et al., 2016; Cheong et al., 2010; Saboo et al., 2015; Madi et al., 2015; Swain et al., 2014 |
| Italy | 1 | - | Ciprofloxacin | 0 | Arosio et al., 2011 |
| Japan | 2 | Nosocomial | Meropenem | 0 | Hagiya et al., 2014 |
| Jordan | 2 | Intake of contaminated water | Gentamicin | 2 | Khalifa et al., 2015 |

**Supplementary Material**

***Chromobacterium violaceum* Pathogenicity: Updates and Insights from Genome Sequencing of Novel *Chromobacterium* Species**

Juliana Helena Batista, José Freire da Silva Neto^*^

^*^Corresponding author: José Freire da Silva Neto, E-mail: jfsneto@usp.br

**Table S1:** Human infection by *Chromobacterium violaceum* from 2010 to 2017.

**Appendix References**

Ansari, S., Paudel, P., Gautam, K., Shrestha, S., Thapa, S., and Gautam, R. (2015). *Chromobacterium violaceum* isolated from a wound sepsis: A case study from Nepal. *Case Rep. Infect. Dis.* 2015:181946. doi:10.1155/2015/181946.

Arosio, M., Raglio, A., Ruggeri, M., Serna-Ortega, P., Morali, L., De Angelis, C., et al. (2011). *Chromobacterium violaceum* lymphadenitis successfully treated in a Northern Italian hospital. *New Microbiol.* 34, 429–432.

Cheong, B. M. (2010). A fatal case of pulmonary *Chromobacterium violaceum* infection in an adult. *Med. J. Malaysia* 65, 148–149.

Bottieau, E., Mukendi, D., Kalo, J. R., Mpanya, A., Lutumba, P., Barbé, B., et al. (2015). Fatal *Chromobacterium violaceum* bacteraemia in rural Bandundu, Democratic Republic of the Congo. *New Microbes New Infect.* 3, 21–23. doi:10.1016/j.nmni.2014.10.007.

Campbell, J. I., Lan, N. P., Qui, P. T., Dung, L. T., Farrar, J. J., and Baker, S. (2013). A successful antimicrobial regime for *Chromobacterium violaceum* induced bacteremia. *BMC Infect. Dis.* 13:4. doi:10.1186/1471-2334-13-4.

Hagiya, H., Murase, T., Suzuki, M., Shibayama, K., Kokumai, Y., Watanabe, N., et al. (2014). *Chromobacterium violaceum* nosocomial pneumonia in two Japanese patients at an intensive care unit. *J. Infect. Chemother.* 20, 139–142. doi:10.1016/j.jiac.2013.10.001.

Kaniyarakkal, V., Orvankundil, S., Lalitha, S. K., Thazhethekandi, R., and Thottathil, J. (2016). *Chromobacterium violaceum* septicaemia and urinary tract infection: Case reports from a tertiary care hospital in South India. *Case Rep. Infect. Dis.*, 1:4. doi:10.1155/2016/6795743.

Karthik, R., Pancharatnam, P., and Balaji, V. (2012). Fatal *Chromobacterium violaceum* septicemia in a South Indian adult. *J. Infect. Dev. Ctries.* 6, 751–755. doi:10.3855/jidc.1866.

Ke, L., An, K. P., Heng, S., Riley, M., Sona, S., Moore, C. E., et al. (2012). Paediatric *Chromobacterium violaceum* in Cambodia: the first documented case. *Trop. Doct.* 42, 178–179. doi:10.1258/td.2012.120054.

Khalifa, S. M., Khaldi, T., Alqahtani, M. M., and Ansari, A. M. (2015). Two siblings with fatal *Chromobacterium violaceum* sepsis linked to drinking water. 2015: bcr2015210987. doi:10.1136/bcr-2015-210987.

Madi, D. R., Vidyalakshmi, K., Ramapuram, J., and Shetty, A. K. (2015). Case report: Successful treatment of *Chromobacterium violaceum* sepsis in a South Indian adult. *Am. J. Trop. Med. Hyg.* 93, 1066–1067. doi:10.4269/ajtmh.15-0226.

Meher-Homji, Z., Mangalore, R. P., Johnson, P. D. R., and Chua, K. Y. L. (2017). *Chromobacterium violaceum* infection in chronic granulomatous disease: A case report and review of the literature. *JMM Case Reports* 4:e005084. doi:10.1099/jmmcr.0.005084.

Pant, N. D., Acharya, S. P., Bhandari, R., Yadav, U. N., Saru, D. B., and Sharma, M. (2017). Bacteremia and urinary tract infection caused by *Chromobacterium violaceum* : Case reports from a tertiary care hospital in Kathmandu, Nepal. *Case Rep. Med.* 2017:7929671 . doi:10.1155/2017/7929671.

Pant, N. D., Sharma, M., and Khatiwada, S. (2015). Asymptomatic bacteriuria caused by *Chromobacterium violaceum* in an immunocompetent adult. *Case Rep. Med.*, 2015:652036. doi:10.1155/2015/652036.

Parajuli, N. P., Bhetwal, A., Ghimire, S., Maharjan, A., Shakya, S., Satyal, D., et al. (2016). Bacteremia caused by a rare pathogen – *Chromobacterium violaceum*: a case report from Nepal. 9, 441–446. doi:  [10.2147/IJGM.S125183](https://dx.doi.org/10.2147%2FIJGM.S125183)

Saboo, A. R., Vijaykumar, R., Save, S. U., and Bavdekar, S. B. (2015). A rare nonfatal presentation of disseminated *Chromobacterium violaceum* sepsis. *J. Microbiol. Immunol. Infect.* 48, 574–577. doi:10.1016/j.jmii.2012.11.002.

Swain, B., Otta, S., Sahu, K. K., Panda, K., and Rout, S. (2014). Urinary tract Infection infection by *Chromobacterium violaceum*. *J. Clin. Diagnostic Res*. 8:DD01–DD02, 6–7. doi:10.7860/JCDR/2014/9230.4703.
